# Integrated computational analysis identifies FABP4, PTGS2, and HPGD as Key molecular targets linking PET microplastic exposure to metabolic dysfunction-associated steatotic liver disease

**DOI:** 10.1371/journal.pone.0354607

**Published:** 2026-07-24

**Authors:** Yi Zhang, Yao Yu, Bin Ge, Dacai Gong, Lan Zheng, Yuanyi Wang, Peng Chen

**Affiliations:** 1 Department of Clinical Laboratory, Pidu District People’s Hospital, The 3RD Affiliated Hospital of Chengdu Medical College, Chengdu, Sichuan, China; 2 Chongqing Key Laboratory of Translational Research for Cancer Metastasis and Individualized Treatment, Chongqing University Cancer Hospital, Chongqing, China; Kwame Nkrumah University of Science and Technology, GHANA

## Abstract

**Background:**

Metabolic dysfunction-associated steatotic liver disease (MASLD) affects 25–38% of the global population, yet the contribution of environmental polyethylene terephthalate (PET) microplastics to its pathogenesis remains unclear. PET microplastics accumulate in the liver at approximately 4.6 particles per gram of tissue and have been implicated in metabolic disturbance, oxidative stress, and inflammation, but their molecular targets and mechanisms in MASLD are not well defined.

**Methods:**

We integrated three GEO microarray cohorts (GSE37031, GSE63067, GSE89632) and performed differential expression analysis, weighted gene co-expression network analysis (WGCNA), and PET target prediction using ChEMBL, PharmMapper, and SwissTargetPrediction. Functional enrichment, protein-protein interaction network analysis, CIBERSORT-based immune deconvolution, molecular docking, and 100 ns molecular dynamics simulations were employed to identify and validate hub genes.

**Results:**

Integration of MASLD transcriptomes and PET target predictions yielded 19 overlapping genes enriched in pathways related to lipid metabolism, fatty acid degradation, glycolysis/gluconeogenesis, and chemical carcinogenesis. Network topology consistently highlighted FABP4, PTGS2, and HPGD as central hub genes. Immune deconvolution revealed MASLD-associated alterations characterized by increased M2 macrophages and γδ T cells, with decreased monocytes, dendritic cells, and naive B cells. PTGS2 and FABP4 expression showed strong correlations with innate immune cells. Molecular docking demonstrated favorable PET binding to all three proteins (–6.3 to –6.9 kcal/mol), and molecular dynamics simulations confirmed stable complexes over 100 ns, with predominantly hydrophobic interactions.

**Conclusions:**

Through integrated bioinformatics analysis and molecular simulation, this study identifies FABP4, PTGS2, and HPGD as potential molecular targets through which PET microplastics may influence lipid metabolism, prostaglandin signaling, and innate immune responses in MASLD. Molecular docking and dynamics simulations suggest favorable binding interactions between PET and these proteins.

## 1. Introduction

Metabolic dysfunction-associated steatotic liver disease (MASLD), formerly known as non-alcoholic fatty liver disease (NAFLD), represents a global health crisis affecting approximately 25–38% of the world’s population [1]. Current projections indicate that MASLD prevalence in adults will exceed 55% by 2040, with 15–38% of individuals with type 2 diabetes developing metabolic dysfunction-associated steatohepatitis (MASH) with clinically significant liver fibrosis [2,3]. This progressive condition encompasses a spectrum ranging from simple hepatic steatosis to MASH, fibrosis, cirrhosis, and ultimately hepatocellular carcinoma. Beyond liver-specific complications, MASLD significantly increases the risk of cardiovascular disease, type 2 diabetes, chronic kidney disease, and extrahepatic cancers, with cardiovascular disease remaining the leading cause of mortality in affected patients [1].

The pathophysiology of MASLD is multifactorial, involving dysregulated lipid metabolism, insulin resistance, oxidative stress, and chronic low-grade inflammation. Oxidative and nitrosative stress are recognized as significant contributors to hepatocellular injury in MASLD and critical drivers of the transition from simple steatosis to MASH. Despite extensive research into metabolic and genetic factors underlying MASLD pathogenesis, the contribution of environmental pollutants—particularly microplastics—to disease development and progression remains poorly characterized [4].

Microplastics, defined as plastic particles smaller than 5 millimeters, have emerged as ubiquitous environmental contaminants with profound implications for human health. Polyethylene terephthalate (PET) is one of the most prevalent microplastic polymers, with global production exceeding 70 million tons annually, accounting for approximately 9% of total plastic consumption and 12% of global solid waste [5]. PET microplastics are pervasive in ecosystems, air, and food sources, and due to their persistence, they can enter biological systems and accumulate in various organs, posing significant health risks [6].

Recent advances in detection methodologies have revealed the alarming extent of human microplastic exposure. Using pyrolysis gas chromatography-mass spectrometry, researchers detected microplastics in all 62 human placental samples analyzed, with concentrations ranging from 6.5 to 790 micrograms per gram of tissue, with polyethylene representing 54% of total plastics detected [7]. Particles smaller than 100 μm can penetrate biological barriers and accumulate in various tissues, including the placenta, blood, and internal organs [8]. A critical finding indicates that microplastics accumulate in the liver at approximately 4.6 particles per gram of tissue, raising concerns about their potential role in hepatic disease pathogenesis [6].

Experimental evidence increasingly supports the hepatotoxic potential of PET microplastics. Chronic exposure to environmentally relevant PET microplastics for 29 weeks in mice led to increased adiposity, hepatomegaly, steatosis, and early-stage fibrosis, with disruption of gut-liver homeostasis [9]. Oral exposure to PET microplastics at doses of 1 mg/day over 42 days resulted in hepatocyte swelling, inflammatory cell infiltration, collagen deposition, and elevated serum transaminases in male mice [10]. High-dose chronic PET microplastic exposure substantially altered intestinal flora diversity, with increased abundances of specific bacterial genera accompanied by elevated lipid metabolites, disrupting metabolic processes and accelerating lipid deposition in the liver [11]. PET breakdown products, including terephthalic acid, promote macrophage polarization toward pro-inflammatory phenotypes and activate the NF-κB signaling pathway, potentially elevating reactive oxygen species production and oxidative stress [12].

Notably, microplastics have been detected in cirrhotic liver tissue but not in healthy liver samples, suggesting potential accumulation in diseased liver states. This observation raises a fundamental question: does hepatic microplastic accumulation represent a causative factor in liver disease progression, or is it a consequence of compromised hepatic clearance mechanisms? While our computational study does not directly resolve this causality question, it takes the essential first step by systematically mapping potential molecular targets through which PET microplastics may contribute to MASLD development [5].

Despite growing evidence of microplastic-mediated hepatotoxicity, the specific molecular targets and signaling pathways through which PET microplastics exert their effects in MASLD remain largely undefined. Traditional toxicological approaches have focused on observational endpoints such as histopathological changes, serum biomarkers, and oxidative stress parameters, but systematic identification of protein targets and mechanistic pathways has been lacking. Network pharmacology and systems biology approaches offer powerful tools to comprehensively map potential molecular interactions and identify key regulatory nodes in disease pathogenesis.

In this study, we employed an integrated computational approach combining target prediction, transcriptomic analysis, weighted gene co-expression network analysis (WGCNA), functional enrichment analysis, protein-protein interaction network construction, molecular docking, and molecular dynamics simulations to systematically identify and validate potential molecular targets through which PET microplastics may contribute to MASLD pathogenesis. By analyzing publicly available MASLD transcriptomic datasets and predicting PET-protein interactions, we identified three hub genes—FABP4, PTGS2, and HPGD—as potential key mediators of PET-associated hepatotoxicity. We further characterized immune infiltration patterns and validated the stability of PET-protein complexes through computational structural biology approaches. Our in silico analyses suggest a potential molecular framework linking PET microplastic exposure to MASLD development and identify putative therapeutic targets that require rigorous experimental validation to establish their role in environmental pollutant-mediated liver disease.

## 2. Materials and methods

### 2.1. Acquisition of PET targets

The molecular structure of PET and Simplified Molecular Input Line Entry System (SMILES) “CC(––O)C1––CC––C(C––C1)C(––O)OCCOC” were obtained from the PubChem database [13]. Potential human protein targets were predicted with three complementary approaches to maximize recall and reduce method-specific bias: (1) ChEMBL (ligand–target bioactivity inference), (2) PharmMapper (reverse pharmacophore mapping), and (3) SwissTargetPrediction (2D/3D similarity and statistical learning) [14]. Predicted targets were merged by UniProt gene symbols, deduplicated, and restricted to Homo sapiens entries.

### 2.2. Dataset acquisition and preprocessing

Three MASLD transcriptomic datasets (GSE37031, GSE63067, and GSE89632) were retrieved from the NCBI GEO database, comprising a total of 38 control and 58 NAFLD samples. To mitigate batch effects across datasets, a two-stage normalization pipeline was implemented: (1) Quantile Normalization: Raw expression matrices were normalized within each dataset to ensure comparable signal distributions. (2) ComBat Harmonization: Inter-dataset batch variations were corrected using the ComBat algorithm from the sva package, which applies parametric empirical Bayes frameworks to adjust batch-specific effects while preserving biological variation between disease and control groups [5]. Principal component analysis (PCA) was performed before and after batch correction to verify effective removal of technical artifacts and improved sample clustering by biological condition. The harmonized expression matrix was used for all downstream analyses [5].

### 2.3. Identification of differentially expressed genes

Differential expression analysis was performed on the harmonized expression matrix using the limma package in R [15]. Linear models were fitted with disease status (MASLD vs. control) as the primary covariate, followed by empirical Bayes moderation of gene-wise variances to enhance statistical power. Differentially expressed genes (DEGs) were identified using the criteria: |log₂ fold change| ≥ 1.5 and adjusted p-value (FDR) < 0.05, with Benjamini-Hochberg correction for multiple testing. Results were visualized through volcano plots and heatmaps.

### 2.4. Weighted Gene Co-Expression Network Analysis (WGCNA)

A scale-free co-expression network was constructed using the WGCNA package [16]. Sample quality control was performed with outlier removal by hierarchical clustering. Optimal soft-thresholding power was determined using the pickSoftThreshold function to achieve scale-free topology (R² > 0.85). Genes were hierarchically clustered based on topological overlap matrix (TOM) with dynamic tree-cutting parameters (minModuleSize = 30, mergeCutHeight = 0.25). Module eigengenes were correlated with MASLD phenotype using Pearson’s correlation (|R| > 0.5, P < 0.05). Genes with high intramodular connectivity (kME > 0.8) in disease-associated modules were prioritized for downstream analysis.

### 2.5. Functional enrichment analysis

Gene Ontology (GO) and Kyoto Encyclopedia of Genes and Genomes (KEGG) pathway enrichment analyses were performed using the clusterProfiler package in R. GO terms were categorized into biological processes (BP), molecular functions (MF), and cellular components (CC). KEGG pathway analysis identified significantly enriched metabolic and signaling pathways. Enrichment significance was determined using hypergeometric tests with Benjamini-Hochberg correction, with adjusted p-value < 0.05 considered statistically significant.

### 2.6. PPI network construction

The STRING database was used to construct protein-protein interaction networks for the overlapping genes between PET targets and MASLD-associated genes. Interactions with a confidence score ≥ 0.4 were included. The resulting network was visualized and analyzed using Cytoscape software. Six topological algorithms implemented in the cytoHubba plugin were applied to identify hub genes: Degree, Closeness, Betweenness, Eigenvector, Edge Percolated Component (EPC), Maximum Neighborhood Component (MNC), and Maximum Clique Centrality (MCC). The MCODE algorithm was used to identify densely connected sub-networks. Genes consistently ranked highly across multiple algorithms were designated as hub genes.

### 2.7. Immune infiltration analysis

To estimate immune composition, bulk RNA-seq profiles were deconvolved with CIBERSORT using the LM22 signature matrix (22 immune cell types). Only samples with CIBERSORT deconvolution p < 0.05 were retained. Relative fractions were compared between groups, and associations between core gene expression and immune fractions were evaluated using Spearman correlation with FDR control.

### 2.8. Molecular docking

Protein structures of core targets were retrieved from the Protein Data Bank, prioritizing high-resolution human crystal structures or high-confidence AlphaFold models where experimental structures were unavailable. Proteins were prepared through removal of crystallographic waters and co-factors, addition of polar hydrogens, and assignment of protonation states at physiological pH (7.4). The PET ligand structure was energy-minimized and protonated accordingly. Molecular docking was conducted using AutoDock Vina with grid boxes centered on predicted or experimentally determined binding pockets. Top-ranked binding poses with free energies ≤ –5.0 kcal/mol were considered indicative of favorable interactions and visually inspected in PyMOL for geometric and chemical plausibility. Selected protein-PET complexes were subjected to 100 ns all-atom molecular dynamics simulations using GROMACS 2022 with the CHARMM36 force field and TIP3P water model under standard temperature and pressure conditions.

### 2.9. Molecular dynamics simulations

To validate the stability of PET-protein complexes, 100 ns all-atom molecular dynamics (MD) simulations were performed using GROMACS (version 2022.3) with the AMBER99SB-ILDN force field. Each complex was solvated in a cubic box with TIP3P water model, maintaining a minimum distance of 1.0 nm from the box edges. The systems were neutralized and physiological ionic strength was achieved by adding 0.15 M NaCl. Energy minimization was performed using the steepest descent algorithm until the maximum force was below 1000 kJ/mol/nm. The systems were equilibrated in two phases: NVT ensemble at 310 K for 100 ps followed by NPT ensemble at 1 bar for 100 ps. Production MD simulations were conducted for 100 ns with a 2 fs time step, saving coordinates every 10 ps. Trajectory analysis included calculation of root-mean-square deviation (RMSD), root-mean-square fluctuation (RMSF), radius of gyration (Rg), solvent-accessible surface area (SASA), and hydrogen bond analysis using GROMACS analysis tools. Principal component analysis (PCA) and free energy landscape calculations were performed to assess conformational dynamics.

### 2.10. Comparative analysis with known hepatotoxicants

To contextualize our findings within the broader field of environmental hepatotoxicants, we performed a comparative analysis with known hepatotoxic compounds associated with MASLD exacerbation. Using the Comparative Toxicogenomics Database (CTD, https://ctdbase.org/), we retrieved chemical-gene interaction data for representative compounds including carbon tetrachloride (CCl₄), acetaminophen (APAP), and bisphenol A (BPA). We compared their known target genes with our 19 overlapping PET-MASLD targets to identify shared and unique molecular signatures (see Supplementary S1 Table).

### 2.11. Statistical analysis

**Fig 1:**
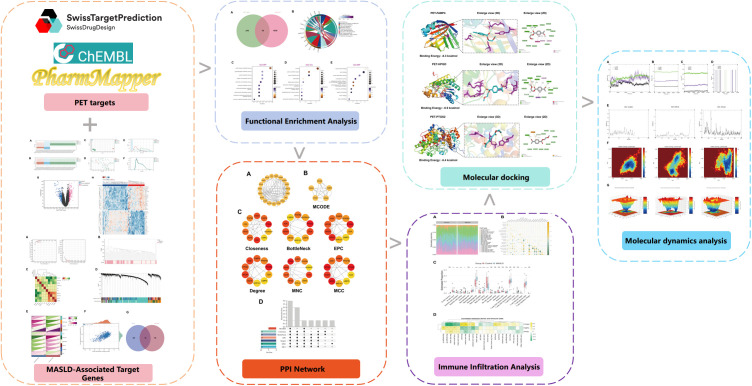
Integrative workflow for the study.

All statistical analyses were performed using R. Continuous variables were compared using Student’s t-test or Wilcoxon rank-sum test as appropriate. Multiple testing corrections were applied using the Benjamini-Hochberg method. Correlation analyses were performed using Pearson or Spearman correlation coefficients. Results with p < 0.05 or adjusted p < 0.05 were considered statistically significant. All visualizations were generated using ggplot2, pheatmap, and ComplexHeatmap package, The flow chart outlining the study is presented in Fig 1 below.

### 2.12. Ethics declarations

Not applicable.

## 3. Results

### 3.1. Identification of potential protein targets of PET

**Fig 2:**
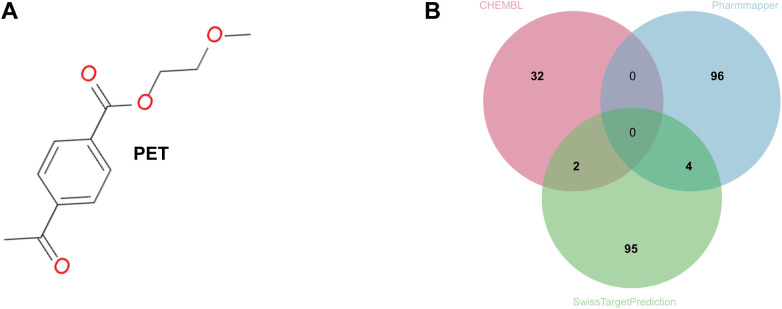
Structure of PET and Prediction of Potential Targets. (A) Chemical structure of PET obtained from the PubChem database. (B) Merged and de-duplicated target sets from ChEMBL, PharmMapper, and SwissTargetPrediction, identifying 229 unique candidate targets.

The standardized chemical structure of polyethylene terephthalate (PET) was retrieved to support subsequent analyses (Fig 2A). Using three target-prediction platforms—ChEMBL, PharmMapper, and SwissTargetPrediction—a comprehensive set of candidate PET-associated proteins was obtained. After merging the outputs from all platforms and removing duplicate entries, a total of 229 non-redundant potential target proteins were identified (Fig 2B). This integrated target set serves as the basis for further functional and bioinformatic analyses.

### 3.2. Integrated processing and differential analysis

**Fig 3:**
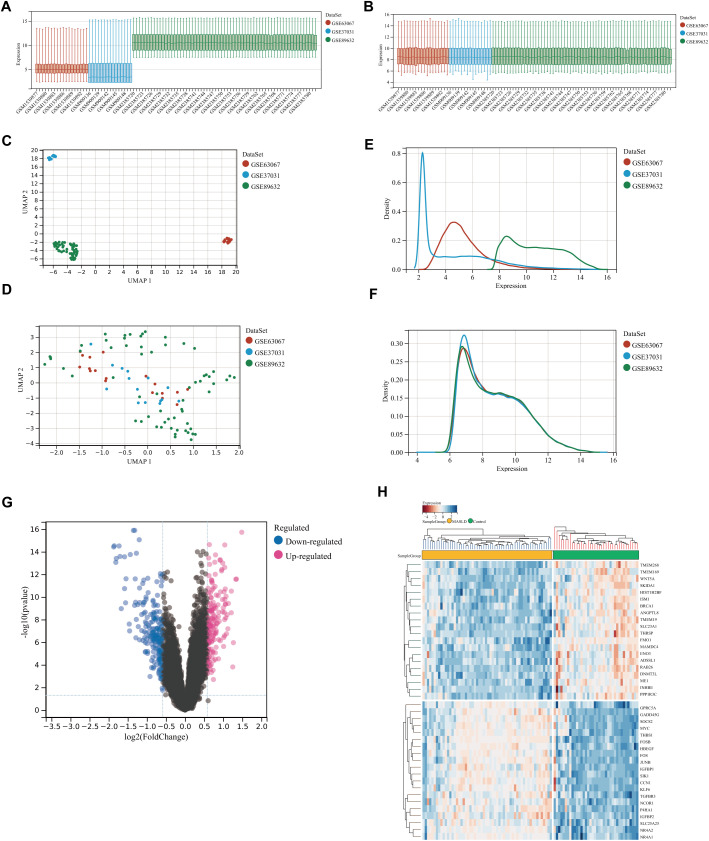
Cross-Dataset Expression Harmonization and DEG Identification（A）Boxplot displaying the distribution of gene expression values across samples from the three datasets prior to normalization and batch correction. (B)Boxplot showing the expression distributions after normalization and batch-effect correction, demonstrating improved consistency across all samples. (C)UMAP plot illustrating sample clustering before batch correction. (D)UMAP plot showing sample distribution after batch correction. (E)Density plot of global gene expression levels before batch-effect correction. (F)Density plot of gene expression distributions after correction. (G)Volcano plot displaying significantly upregulated and downregulated genes. (H)Heatmap of the top differentially expressed genes.

After merging and batch-correcting the GSE37031, GSE63067, and GSE89632 datasets, the overall expression distributions across samples became consistent (Fig 3A–B). UMAP visualization showed that samples no longer clustered by dataset source after correction, indicating a uniform distribution across all samples (Fig 3C–D). Density plots further confirmed the alignment of global expression patterns following correction (Fig 3E–F). Based on the integrated expression matrix, the limma package was used for differential analysis, and the Benjamini–Hochberg method was used for multiplex test correction. The threshold is set to |log₂FC| ≥ 2 and p < 0.05. A total of 357 differential genes were obtained, of which 153 were up-regulated and 204 were down-regulated. (Fig 3G). In the differential gene heat map, the top 40 core differential genes sorted by significance were selected. These genes exhibited highly consistent expression pattern differences between samples, with the disease group forming a well-defined bibranched cluster structure with the control group (Fig 3H). The expression of the top 40 genes showed stable typing characteristics after Z-score normalization.

### 3.3. Construction of co-expression modules and screening of MASLD-associated hub genes

**Fig 4:**
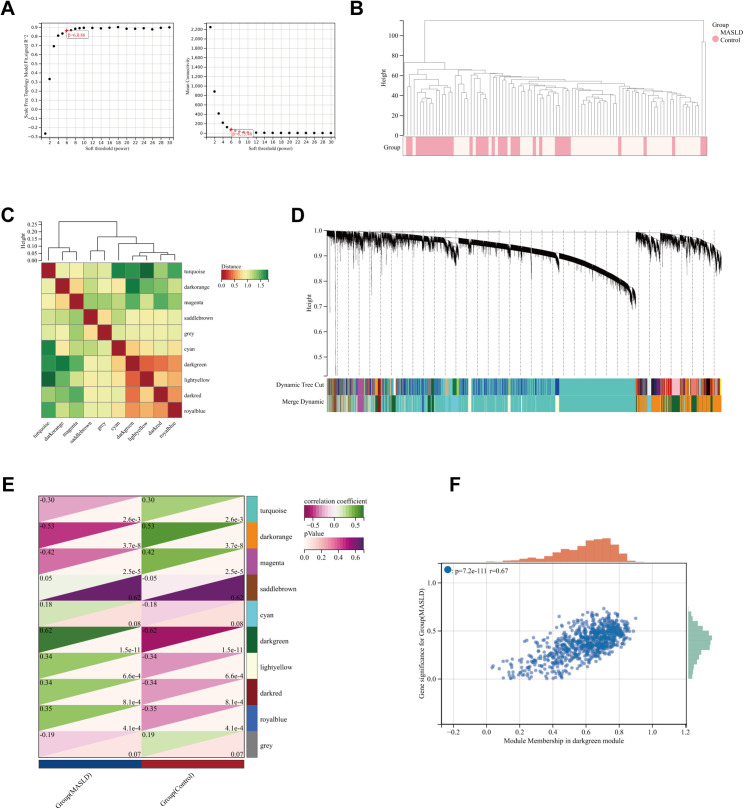
Construction of the weighted gene co-expression network and identification of MASLD-associated modules. (A) Soft-thresholding power selection for WGCNA. (B) Sample clustering dendrogram based on hierarchical clustering. (C) Module eigengene clustering heatmap showing similarity among modules. (D) Gene clustering dendrogram derived from the topological overlap matrix (TOM). (E) Heatmap of module–trait correlations. (F) Correlation between gene significance (GS) and module membership (MM) within the darkgreen module. (G) Venn diagram illustrating the overlap between differentially expressed genes (DEGs) and genes in the key WGCNA module.

To determine the appropriate soft-thresholding power for constructing a scale-free co-expression network, the scale-free topology fit index and mean connectivity were evaluated across a range of β values. A soft threshold of β = 6 was selected, as it achieved a scale-free topology fit of R² = 0.86 and exhibited a reasonable mean connectivity trend (Fig 4A). Using this threshold, sample clustering was first performed to assess potential outliers, confirming that all samples were suitable for downstream analysis (Fig 4B). Subsequently, genes were clustered based on the topological overlap matrix (TOM), generating a series of initial gene modules through dynamic tree cutting (Fig 4D). Modules with highly similar eigengenes were then merged, yielding the final module set, including turquoise, darkorange, magenta, saddlebrown, grey, cyan, darkgreen, lightyellow, darkred, and royalblue (Fig 4C). Module–trait correlation analysis identified the darkgreen module as the most significantly associated with MASLD, showing a strong positive correlation (r = 0.62; p = 1.5 × 10 ⁻ ¹¹) (Fig 4E). Further assessment of the darkgreen module demonstrated a strong positive correlation between gene significance and module membership (r = 0.67; p = 7.2 × 10 ⁻ ¹¹¹) (Fig 4F), indicating that genes with higher intramodular connectivity were also more strongly associated with MASLD. Intersection analysis between the differentially expressed genes (DEGs) and the genes contained within the WGCNA-identified key module showed that the WGCNA module set included 736 genes, while 265 DEGs were identified through differential expression analysis, and their overlap yielded 95 intersecting genes, representing the candidate MASLD-related hub genes obtained from integrating co-expression network analysis with differential expression results (Fig 4G).

### 3.4. Enrichment analysis of PET-associated disease targets in HCC

**Fig 5:**
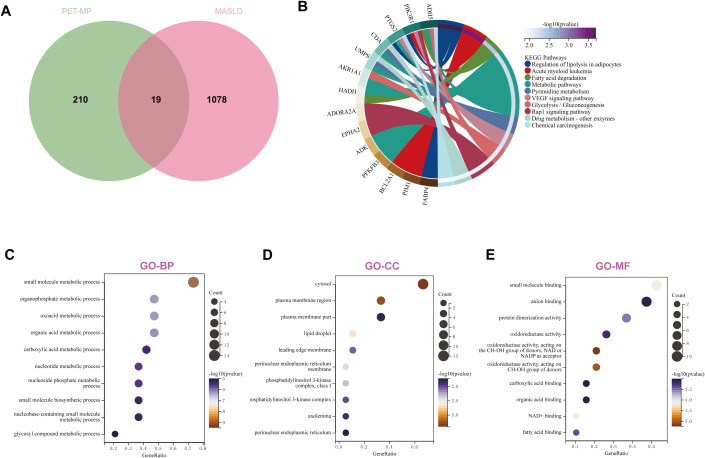
Overlap and Enrichment Analysis of PET and MASLD Targets. (A)Venn diagram showing 19 shared targets.(B)Chord diagram of the top enriched KEGG pathways for the 19 core genes.(C-E) Bubble chart of the top enriched GO terms.

To explore the biological implications of PET exposure in the context of MASLD, an intersection analysis was performed between the predicted PET target proteins and the identified MASLD-related genes. This comparison yielded 19 overlapping genes, representing potential key targets mediating PET-associated hepatocarcinogenesis (Fig 5A). To evaluate the potential specificity of PET-associated targets, we compared the 19 overlapping genes with targets of well-known hepatotoxicants from the CTD database (see Supplementary S1 Table). Carbon tetrachloride (CCl₄), a classic hepatotoxicant, overlapped with only 3 of the 19 genes and did not target FABP4 or HPGD. Acetaminophen (APAP) and bisphenol A (BPA) overlapped with 11 and 12 genes, respectively, and both targeted all three hub genes (FABP4, PTGS2, and HPGD). Notably, PET showed the broadest overlap (19 genes), suggesting that PET may exert more extensive effects on MASLD-related pathways compared to these other hepatotoxicants. To investigate the biological functions associated with the MASLD-related hub genes, Gene Ontology (GO) and Kyoto Encyclopedia of Genes and Genomes (KEGG) enrichment analyses were performed.KEGG enrichment analysis further indicated that the hub genes participated in several key metabolic and signaling pathways, including regulation of lipolysis in adipocytes, acute myeloid leukemia，fatty acid degradation, metabolic pathways, pyrimidine metabolism, glycolysis/gluconeogenesis, VEGF signaling, Rap1 signaling, drug metabolism, and chemical carcinogenesis(Fig 5B). GO enrichment analysis indicated that the hub genes were predominantly associated with several top-ranked pathways of high biological relevance. In the BP category, the leading enriched terms were primarily related to small-molecule metabolic processes, including small-molecule metabolic process, organophosphate metabolic process, and organic/carboxylic acid metabolic process, suggesting that these genes play central roles in energy metabolism and substrate conversion. Within the MF category, the highest-value terms were enriched in small-molecule binding and oxidoreductase activity, reflecting the importance of these gene products in substrate recognition and redox regulation. For the CC category, the top enriched cellular localizations included the cytosol, plasma membrane region, and lipid droplet, as well as membrane-associated complexes such as the phosphatidylinositol 3-kinase complex (class I). These findings imply that the hub genes may function at the intersection of metabolic regulation and membrane-related signal transduction. Taken together, the top-ranked GO terms consistently point toward roles in small-molecule metabolism, redox regulation, and membrane-associated functional compartments, underscoring the potential involvement of these hub genes in cellular metabolic reprogramming.

### 3.5. Construction of the PPI network and extraction of key hub nodes

**Fig 6:**
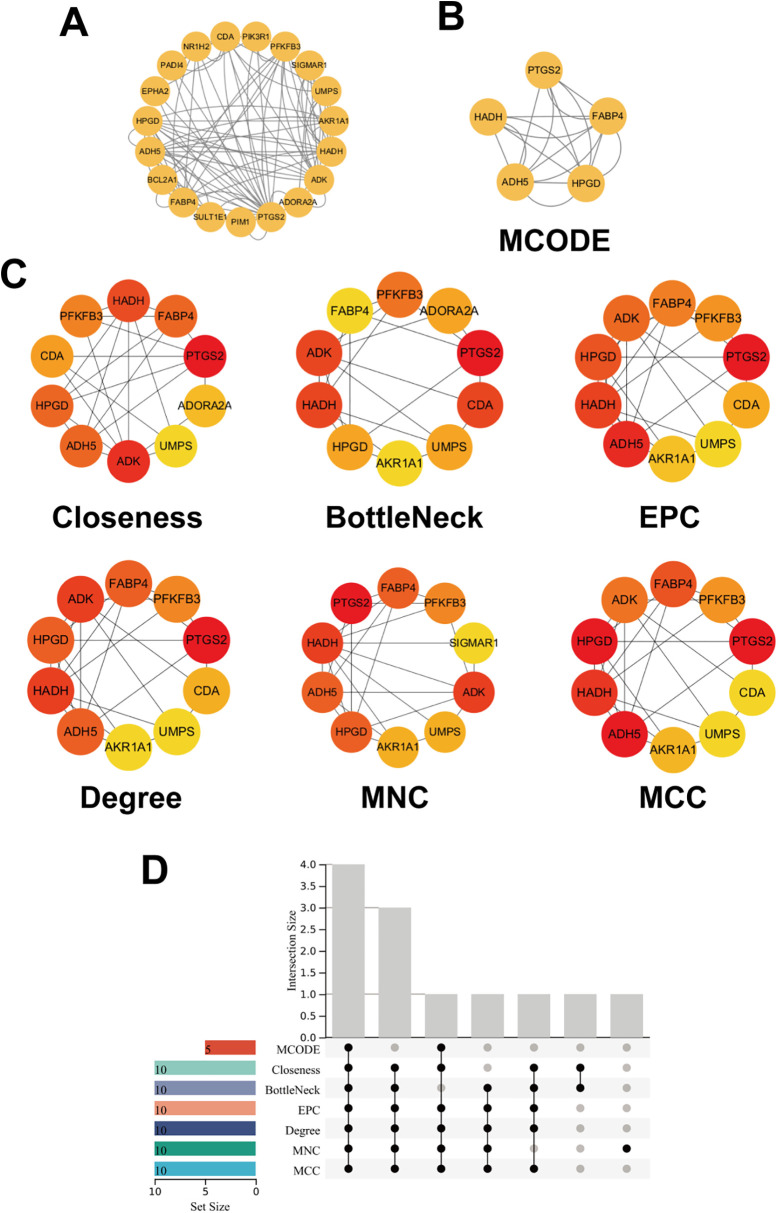
PPI Network and Hub Gene Screening among 19 Common Targets. (A)Cytoscape visualization of the PPI network consisting of 19 nodes and 92 edges. (B)MCODE-identified functional module extracted from the PPI network. (C)Top 10 core targets ranked by six CytoHubba centrality algorithms, including Closeness, BottleNeck, EPC,Degree, MNC,MCC; darker colors indicate higher ranking. (D)UpSet plot showing the overlap of hub genes identified by different centrality algorithms, highlighting PTGS2, HPGD, and FABP4.

To elucidate the interaction relationships among the candidate targets, a Protein–Protein Interaction (PPI) network was constructed and visualized in Cytoscape (Fig 6A). The curated network contained 19 nodes and 92 edges, exhibiting a compact and highly interconnected topology that reflects substantial functional coordination among the included proteins. To further resolve the internal organization of the network, a densely aggregated functional submodule was identified using the MCODE plugin in Cytoscape (Fig 6B). This subnetwork displayed markedly increased connectivity, suggesting the presence of a structurally cohesive core module. Subsequently, six topological centrality algorithms implemented in the CytoHubba plugin of Cytoscape—including closeness, degree, eigenvector, edge percolation centrality (EPC), maximum clique centrality (MCC), and maximum neighborhood component (MNC)—were applied to quantify the structural importance of each node within the network (Fig 6C). These indices characterized node contributions to network cohesion and information flow from multiple topological perspectives. An UpSet plot was then generated to compare the overlap among hub genes identified by the different centrality algorithms (Fig 6D). The results revealed that PTGS2, HPGD, and FABP4 were consistently highlighted across multiple algorithms, indicating their stable and prominent centrality within the PPI network.

### 3.6. Immune cell infiltration analysis

**Fig 7:**
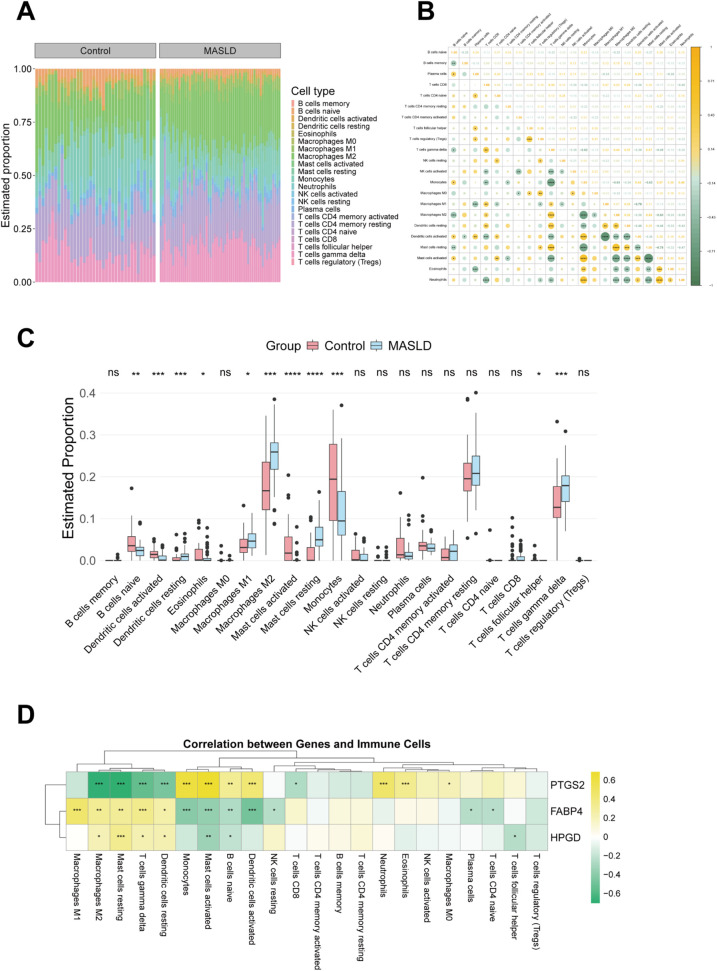
Immune infiltration landscape and gene–immune associations in MASLD. (A) Stacked bar plot showing per-sample proportions of 22 immune cell subsets (CIBERSORT) in Control and MASLD. (B) Correlation matrix (heatmap) of pairwise correlations among the 22 immune cell subsets with significance annotations. (C) Box plots comparing estimated proportions between groups for each immune cell subset. Statistical significance was assessed using the Wilcoxon rank-sum test: `ns` p ≥ 0.05, `*` p < 0.05, `**` p < 0.01, `***` p < 0.001.” (D) Heatmap showing correlations between three core genes and estimated immune cell proportions, with significance indicated.

In the analysis of immune infiltration composition in liver tissue (using stacked bar charts), significant individual differences were observed in both the Control and MASLD groups. Overall, macrophages (primarily M2 type) and T cells (mainly CD4 memory resting) predominated in most samples. In contrast, B cells (naive and memory) and plasma cells were relatively low in proportion across all samples. Mast cells (both activated and resting) and eosinophils were found to occupy a lower proportion in the majority of samples. NK cells (resting and activated) and neutrophils showed moderate to low proportions in some samples, displaying certain fluctuations. Visually, in the MASLD group, a prominent bandwidth of M2 macrophages and neutrophils/monocytes was observed in some samples, whereas in the Control group, the bandwidth of CD4 memory resting T cells and NK cells was more prominent(Fig 7A). The correlation plot of immune infiltration revealed strong mutual exclusion between the “resting-activated” states of similar cell types. For instance, dendritic cells and mast cells showed a strong negative correlation between their resting and activated states (r ≈ −0.78), and M1 and M2 macrophages also exhibited a significant negative correlation (r ≈ −0.78). Moreover, NK cell activation was moderately to strongly negatively correlated with monocytes (r ≈ −0.65), but positively correlated with activated mast cells, neutrophils, and eosinophils (r ≈ 0.33–0.57). NK activation also showed a negative correlation with γδ T cells (r ≈ −0.62). Apart from these relationships, the correlations between most T cell subpopulations and their association with myeloid cells were close to zero and weakly significant, suggesting that the primary covariation in immune infiltration is concentrated in the myeloid/intrinsic immune components and the opposing changes between the “resting-activated” states. Further analysis showed that the correlation between the three genes and immune infiltration was most prominent in myeloid and allergy-related cells. Specifically, PTGS2 showed significant positive correlations with monocytes, neutrophils, eosinophils, activated mast cells, and activated dendritic cells, while negatively correlating with resting mast cells, resting dendritic cells, M2 macrophages, and γδ T cells. Most T cell subpopulations showed weak associations with PTGS2. FABP4 was positively correlated with both M1 and M2 macrophages but negatively correlated with monocytes, activated mast cells, activated dendritic cells, and naive B cells. It showed no significant correlation with other lymphocytes. HPGD had a relatively small overall effect, primarily negatively correlating with both resting and activated mast cells, and showing a mild negative correlation with M2 macrophages, γδ T cells, and naive B cells(Fig 7B). In summary, PET-MASLD-associated immune infiltration changes were more closely related to alterations in the innate immunity/myeloid axis, with relatively limited associations with most T cell subpopulations(Fig 7C). When comparing the MASLD group to the Control group, significant remodeling of the immune composition was observed, primarily involving changes in myeloid immune cells: M2 macrophages were significantly increased, while M1 macrophages showed a slight increase; monocytes significantly decreased; dendritic cells (both activated and resting) showed a decrease; mast cells underwent phenotypic changes, with a decrease in activated mast cells and an increase in resting mast cells. Simultaneously, γδ T cells were significantly elevated, and Tfh cells showed a slight increase. Naive B cells and eosinophils were significantly decreased in the MASLD group. Other cells, such as NK cells, neutrophils, CD4 memory/naive T cells, CD8 T cells, and Tregs, showed no significant changes, suggesting that the major immune alterations in MASLD are centered on the monocyte-macrophage, dendritic cell, and mast cell axes, with an accompanying rise in γδ T cells(Fig 7D).

### 3.7. Molecular docking analysis of PET with hub gene proteins

**Fig 8:**
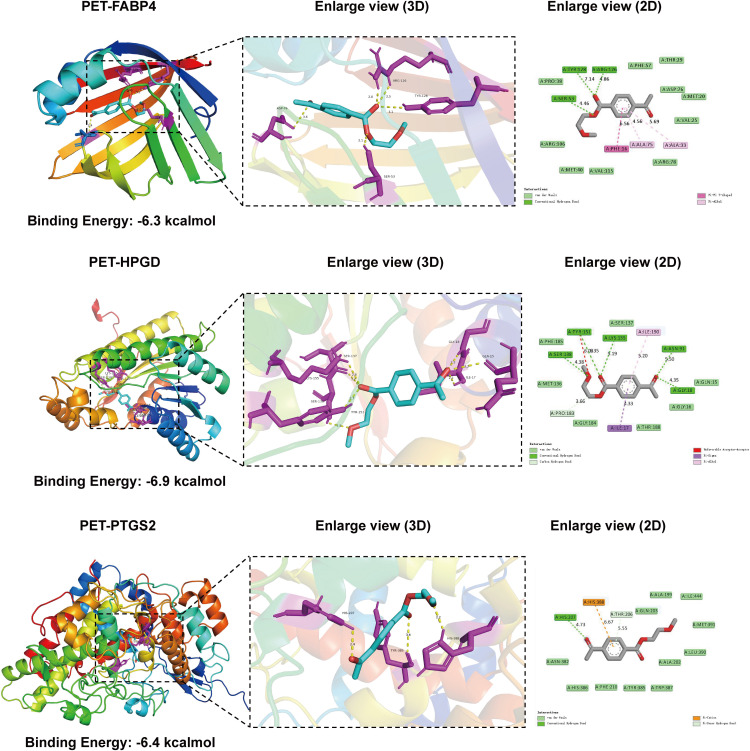
Molecular docking poses and binding free energies between PET and the three core target proteins.

To verify the direct binding of PET to the selected targets, molecular docking was performed for FABP4, HPGD, and PTGS2 (Fig 8). The results showed that PET formed stable complexes within the binding cavities of all three proteins, with binding energies of –6.3 kcal/mol for FABP4, –6.9 kcal/mol for HPGD, and –6.4 kcal/mol for PTGS2, yielding an overall affinity order of HPGD > PTGS2 > FABP4. The corresponding 3D/2D representations illustrate the spatial conformations of the complexes and the key interaction types, including conventional hydrogen bonds, carbon–hydrogen bonds, van der Waals contacts, and π–π/π–alkyl interactions. Specifically, in FABP4, PET engages in hydrogen bonding with Ser53, Tyr128, and Arg126, is stabilized by multiple hydrophobic residues, and further interacts through aromatic contacts with Phe16 and Ala33. In HPGD, a network of hydrogen bonds involving Ser138, Tyr151, and Lys155, together with π-related interactions between the aromatic core of PET and Ile17/Ile190, and extensive van der Waals contacts, contribute to the highest binding affinity observed among the three proteins. In PTGS2, PET forms hydrogen bonds and hydrogen bond–assisted interactions with His207, Thr206, and Gln203, and establishes a prominent π–cation interaction with His388, while being tightly enclosed by hydrophobic residues such as Met391, Leu390, and Ala202. Collectively, the combination of hydrogen bonding, hydrophobic encapsulation, and aromatic interactions underlies the stable binding of PET to all three targets.

### 3.8. Molecular dynamics simulation validation of PET-hub protein complex stability

**Fig 9:**
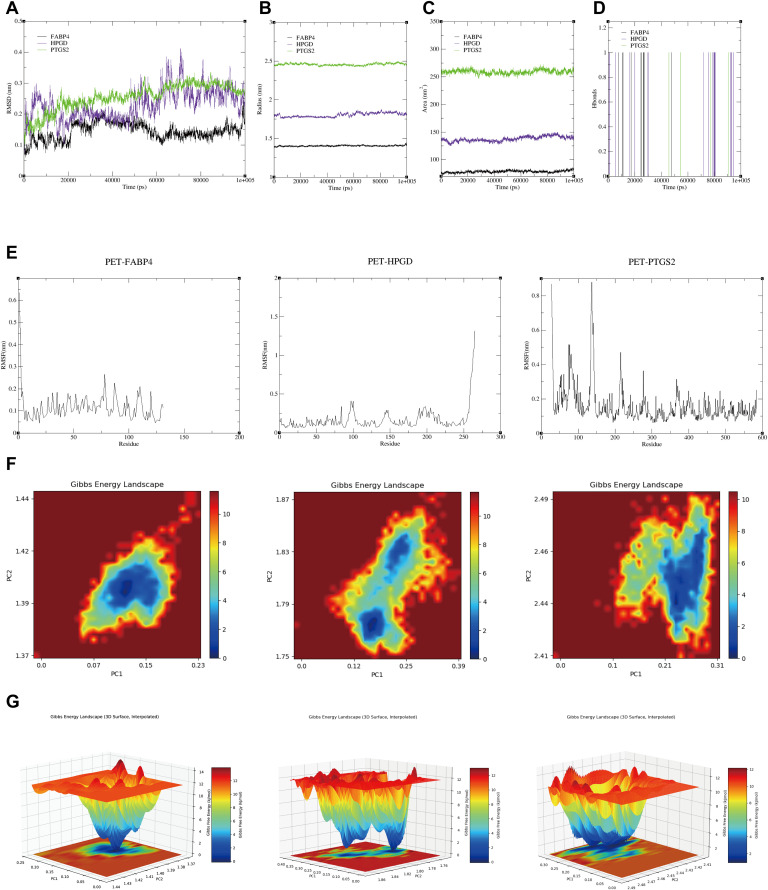
Molecular Dynamics Simulation Analysis of PET-Hub Protein Complexes. (A) RMSD time evolution curves of PET-FABP4, PET-HPGD, and PET-PTGS2 complexes during 100 ns MD simulation. (B) Temporal variation of radius of gyration (Rg) for the three complex systems. (C) Time evolution of solvent accessible surface area (SASA). (D) Variation of protein-ligand hydrogen bond numbers over simulation time. (E) RMSF distribution plots for each residue in the three complex systems, from left to right: PET-FABP4, PET-HPGD, and PET-PTGS2. (F) Two-dimensional Gibbs free energy landscape based on the first two principal components, with colors from blue to red representing free energy from low to high (unit: kJ/mol). (G) Three-dimensional Gibbs free energy surface plot, with Z-axis representing free energy height.

To validate the stability of binding conformations obtained from molecular docking under dynamic conditions, we performed 100 ns all-atom molecular dynamics simulations on three complex systems (PET-FABP4, PET-HPGD, PET-PTGS2) and systematically evaluated their dynamic behavioral characteristics through multiple structural parameters. RMSD analysis (Fig 9A) revealed the conformational evolution patterns of the three complex systems: PET-FABP4 rapidly completed conformational adjustment during the initial phase (approximately 0–20 ns), after which RMSD stabilized in the 0.10–0.18 nm range with minimal fluctuation amplitude; PET-HPGD completed initial relaxation within the first 10–15 ns, then RMSD remained primarily in the 0.20–0.35 nm range with occasional spikes approaching 0.4 nm; PET-PTGS2 maintained a range of 0.22–0.32 nm after equilibration, with overall levels higher than FABP4 but relatively stable fluctuations. All three systems reached stable states in the late simulation phase, with RMSD values predominantly below the 0.4 nm threshold. RMSF analysis (Fig 9E) further revealed residue flexibility distribution patterns: the PET-FABP4 complex exhibited overall low residue flexibility, with RMSF values primarily distributed in the 0.06–0.18 nm range, showing a local peak (0.25–0.27 nm) only at approximately residue 80; most residues in the PET-HPGD complex had RMSF values in the 0.08–0.25 nm range, but an extremely high flexibility peak (1.2–1.3 nm) appeared in the C-terminal 255–270 region; the overall RMSF of the PET-PTGS2 complex was 0.10–0.25 nm, with the maximum peak (0.8–0.9 nm) detected in the 100–160 region. Radius of gyration (Rg) analysis (Fig 9B) showed that the Rg values of all three complex systems remained stable throughout the simulation: PET-FABP4 ranged from 1.45–1.55 nm (mean approximately 1.45 nm), PET-HPGD from 1.75–1.95 nm (mean approximately 1.88 nm), and PET-PTGS2 from 2.40–2.55 nm (mean approximately 2.48 nm), indicating that ligand binding did not cause significant changes in the overall protein folding state. SASA analysis (Fig 9C) showed that the solvent-exposed surface areas of the three complex systems remained overall stable after equilibration: PET-FABP4 maintained primarily 75–85 nm² (mean approximately 80 nm²), PET-HPGD 130–147 nm² (mean approximately 140 nm²), and PET-PTGS2 255–268 nm² (mean approximately 261–263 nm²). Hydrogen bond statistical analysis (Fig 9D) revealed that protein-ligand hydrogen bond counts for all three complexes showed discrete 0/1 distributions, remaining at 0 for most of the simulation time, with transient hydrogen bond formation events observed only at isolated moments, suggesting that the binding stability of these complexes may depend more on hydrophobic interactions and van der Waals forces rather than polar interactions. Principal component analysis (PCA) and Gibbs free energy landscape (Figs 9F–G) showed: PET-FABP4 formed a single and compact energy basin in PC1–PC2 space, with the three-dimensional energy surface presenting a deep and steep funnel-like feature; the energy landscape of PET-HPGD was more dispersed, with two low-energy basins appearing along the principal component direction; PET-PTGS2 formed an elongated low-energy valley in the PC1 ≈ 0.20–0.30, PC2 ≈ 2.43–2.48 region, containing multiple local energy minima. Although all three systems exhibited funnel-shaped energy landscapes, the deepest and most concentrated energy funnel appeared in the PET-FABP4 system, again confirming its superior dynamic stability and conformational homogeneity. In summary, molecular dynamics simulation results demonstrate that all three PET-protein complexes can maintain stable binding conformations on the 100 ns timescale, with PET-FABP4 exhibiting optimal structural stability and the most compact energy landscape, providing a reliable theoretical foundation for subsequent experimental validation.

## 4. Discussion

Microplastics have been detected in multiple human biological matrices, with the liver showing relatively high accumulation levels at approximately 4.6 particles per gram of tissue [17].Recent studies demonstrate that chronic PET microplastic exposure can disrupt gut-liver homeostasis and contribute to hepatic steatosis [18].Meta-analyses of vertebrate studies confirm that microplastics can damage the liver through multiple mechanisms, including morphological alterations, oxidative stress induction, intracellular toxicity, biotransformation disruption, and lipid metabolism disturbance [19].Notably, microplastics have been detected in cirrhotic liver tissue but not in healthy liver samples, suggesting potential accumulation in diseased liver states. This finding raises important questions about whether hepatic microplastic accumulation represents a causative factor in liver disease progression or a consequence of compromised hepatic clearance mechanisms. Our study addresses this gap by systematically mapping potential molecular targets through which PET may contribute to MASLD development [20].

FABP4 emerged as the most clinically relevant target.FABP4 serves as a transport-regulatory protein connecting lipid trafficking between adipose tissue and liver, and represents a validated therapeutic target in MASLD with demonstrated roles in lipid metabolism regulation [21]. FABP4 is secreted from adipocytes in response to lipolysis and elevated circulating FABP4 levels correlate with obesity, metabolic syndrome, and insulin resistance. Our enrichment analyses revealed that the 19 overlapping genes are significantly enriched in metabolic pathways including regulation of lipolysis in adipocytes, fatty acid degradation, and glycolysis/gluconeogenesis—processes in which FABP4 plays a central coordinating role. The identification of FABP4 as a potential PET target suggests that environmental plastic exposure may interfere with normal lipid trafficking mechanisms, potentially exacerbating metabolic dysfunction. PTGS2 represents the inflammatory component of MASLD pathogenesis. PTGS2 is commonly known as COX-2. It is the rate-limiting enzyme that converts arachidonic acid into prostaglandins (such as PGE₂) and is expressed during inflammatory processes [22].Our enrichment analysis revealed that the hub genes participated in VEGF signaling and Rap1 signaling pathways, both of which can be influenced by COX-2-derived prostaglandins.Genetic polymorphisms of COX2 have been shown to modify the association of environmental toxicants like bisphenol A with abnormal liver function, suggesting that oxidative stress-related genes play a critical role in mediating chemical-induced hepatotoxicity [23].This mechanistic parallel supports our hypothesis that PET microplastics may similarly interact with PTGS2 to exacerbate oxidative stress and inflammatory responses in MASLD. HPGD (15-hydroxyprostaglandin dehydrogenase) emerged as a critical regulatory node in prostaglandin metabolism [24]. HPGD catalyzes the oxidation of prostaglandin E2 to the anti-inflammatory 15-keto-PGE2, which serves as an endogenous ligand for peroxisome proliferator-activated receptor-gamma (PPAR-γ) [24,25]. Inhibition of 15-PGDH has been shown to ameliorate MASH-associated apoptosis and fibrosis in mice, with treated animals showing significant decreases in oxidative stress, inflammation, and improved insulin resistance (Recent 2025 study). However, the role of HPGD in MASLD appears context-dependent [26]. Downregulation of 15-PGDH enhances MASH-HCC development via fatty acid-induced T-cell exhaustion, with PGE2 accumulation promoting hepatocyte proliferation [25]. Transgenic mice with targeted hepatic 15-PGDH expression showed lower levels of liver enzymes, less tissue damage, and reduced inflammatory cytokine production in acute liver injury models [24]. Our molecular docking and dynamics simulations demonstrating stable PET-HPGD binding (–6.9 kcal/mol) suggest that PET exposure may interfere with normal prostaglandin catabolism, potentially disrupting the delicate balance between pro-inflammatory PGE2 and anti-inflammatory 15-keto-PGE2.

Our immune infiltration analysis revealed significant alterations in the hepatic immune microenvironment, with increased M2 macrophages and decreased M1 macrophages in MASLD samples(14). This finding aligns with recent microplastics research [27]. Oral ingestion of microplastics in mice increased infiltration of natural killer cells and macrophages to non-parenchymal liver cells while reducing B cell infiltration, with these processes regulated via the NF-κB signaling pathway [28]. Long-term oral microplastic intake in mice revealed increased macrophage infiltration in the liver, significantly raising the percentage of M1 macrophages while decreasing M2 macrophages [29]. Polystyrene microplastics induced liver inflammation and promoted the formation of macrophage extracellular traps, which subsequently amplified inflammation in hepatocytes [30]. Microplastics polarized hepatic macrophages to pro-inflammatory M1 type and facilitated extracellular trap formation, with Kupffer cells playing a central role in lipid metabolism responses to microplastic-induced fat overload.

The intersection of our three hub genes—FABP4, PTGS2, and HPGD—with immune cell infiltration patterns suggests a coordinated mechanism whereby PET microplastics may disrupt lipid-prostaglandin-immune axes. Our correlation analysis demonstrated that PTGS2 showed significant positive correlations with monocytes, neutrophils, eosinophils, and activated mast cells, while FABP4 was positively correlated with both M1 and M2 macrophages but negatively correlated with monocytes and activated mast cells. These associations suggest that PET-mediated perturbations of these molecular targets could fundamentally reshape the hepatic immune landscape.

From a structural biology perspective, our molecular dynamics simulations provide compelling evidence for the physical basis of these toxicities. The RMSD and Radius of Gyration (Rg) analyses indicated that the PET-protein complexes, particularly PET-FABP4, maintained a rigid and stable conformation over 100 ns. Notably, the interaction analysis highlighted the dominance of hydrophobic interactions and van der Waals forces over hydrogen bonding in maintaining complex stability. This is consistent with the chemical nature of polyethylene terephthalate. The solvent-accessible surface area (SASA) results further imply that PET binding might occlude functional surfaces of these proteins, potentially hindering their interaction with downstream effectors or natural substrates. This structural stability suggests that once PET microplastics accumulate in hepatocytes or Kupffer cells, they may form persistent complexes with these metabolic and inflammatory regulators, leading to long-term functional impairment rather than transient interference.

Several limitations of our study should be acknowledged. First, our analyses relied on computational prediction methods and publicly available transcriptomic datasets, which require experimental validation in cell culture and animal models. Second, the molecular docking and dynamics simulations, while indicating favorable binding energetics, do not account for the complex in vivo environment including competitive binding, metabolic transformation of PET particles, and the presence of other cellular factors. Third, the immune infiltration analysis used deconvolution algorithms applied to bulk RNA-seq data rather than single-cell sequencing, which limits resolution of immune cell subpopulations. Fourth, dose-response relationships and chronic versus acute exposure scenarios were not addressed in our computational framework.

Future investigations should prioritize several research directions. Experimental validation of PET-protein interactions using techniques such as surface plasmon resonance, isothermal titration calorimetry, and co-immunoprecipitation is essential. Cell-based assays examining PET effects on FABP4-mediated lipid trafficking, PTGS2-dependent prostaglandin synthesis, and HPGD-catalyzed prostaglandin degradation would provide mechanistic insights. Animal studies using PET microplastic exposure models with tissue-specific knockout of these hub genes would establish causality. While we compared PET-associated targets with known hepatotoxicants at the gene level, comprehensive comparative immune infiltration analysis and molecular docking for other compounds were not performed due to data availability and scope constraints. Future studies should extend these comparisons to further contextualize the unique vs. shared mechanisms of PET microplastics relative to other environmental hepatotoxicants Finally, clinical studies examining associations between microplastic exposure levels, expression of these hub genes, and MASLD severity could translate these findings to human populations.

## 5. Conclusion

This study suggests that PET microplastics may affect MASLD pathogenesis by targeting FABP4, PTGS2, and HPGD. Through integrated transcriptomic analysis and network pharmacology, we identified overlapping genes between predicted PET targets and MASLD-associated transcripts, enriched in lipid metabolism, prostaglandin signaling, and inflammatory pathways. Molecular docking simulations showed that PET has significant binding affinity to these three hub proteins, with binding stability confirmed by molecular dynamics simulations. Immune infiltration analysis revealed correlations between hub gene expression and macrophage populations in MASLD tissues. These computational results provide a basis for in-depth experimental study of PET-induced MASLD mechanisms. Future studies should focus on the dose-response relationship between PET exposure and metabolic liver dysfunction, validate PET-protein binding through biophysical assays, and explore potential interventions to mitigate the adverse effects of microplastics on liver health.

## Supporting information

S1 Table Comparison of PET-assoclated targets with known hepatotoxicants. (DOCX)
