# Supplementary material for: Integrated computational analysis identifies FABP4, PTGS2, and HPGD as Key molecular targets linking PET microplastic exposure to metabolic dysfunction-associated steatotic liver disease
Source: PLoS One. 2026 Jul 24;21(7):e0354607. doi: 10.1371/journal.pone.0354607 (PMC13399308; doi:10.1371/journal.pone.0354607)
Supplement: S1 Table — (DOCX) [file pone.0354607.s001.docx]

**Supplementary Table S1. Comparison of PET-assoclated targets with known hepatotoxicants**

| **Compound** | **Total targets (from CTD)** | **Overlap with 19 PET-MASLD genes** | **Key overlapping genes** | **Includes FABP4?** | **Includes PTGS2?** | **Includes HPGD?** |
| --- | --- | --- | --- | --- | --- | --- |
| Carbon tetrachloride (CCl₄) | 27 | 3 | PTGS2 | No | Yes | No |
| Acetaminophen (APAP) | 7477 | 11 | PTGS2, HPGD, FABP4 | Yes | Yes | Yes |
| Bisphenol A (BPA) | 7239 | 12 | PTGS2, HPGD, FABP4 | Yes | Yes | Yes |
| PET | 229 | 19 | PTGS2, HPGD, FABP4 | Yes | Yes | Yes |

Comparison of PET-associated predicted targets with known hepatotoxicants (carbon tetrachloride, acetaminophen, and bisphenol A) retrieved from the Comparative Toxicogenomics Database (CTD). The table shows total known targets for each compound, the number of overlapping genes with the 19 PET-MASLD overlapping genes, key overlapping genes, and whether each of the three hub genes (FABP4, PTGS2, HPGD) is included. PET targets were predicted using ChEMBL, PharmMapper, and SwissTargetPrediction.
